# Mind the gaps: a qualitative study of perceptions of healthcare professionals on challenges and proposed remedies for cervical cancer help-seeking in post conflict northern Uganda

**DOI:** 10.1186/1471-2296-14-193

**Published:** 2013-12-17

**Authors:** Amos D Mwaka, Henry R Wabinga, Harriet Mayanja-Kizza

**Affiliations:** 1 Department of Medicine, School of Medicine, College of Health Sciences, Makerere University, P.O Box 7072, Kampala, Uganda; 2 Kampala Cancer Registry, Department of Pathology, School of Biomedical Sciences, College of Health Sciences, Makerere University, Kampala, Uganda

**Keywords:** Barriers to care, Cervical cancer, Northern Uganda, Healthcare professionals

## Abstract

**Background:**

There are limited data on perceptions of health professionals on challenges faced by cervical cancer patients seeking healthcare in the developing countries. We explored the views of operational level health professionals on perceived barriers to cervical screening and early help–seeking for symptomatic cervical cancer and the proposed remedies to the challenges.

**Methods:**

Fifteen key informant interviews were held with health professionals including medical directors, gynecologists, medical officers, nurses and midwives in the gynecology and obstetrics departments of two hospitals in northern Uganda during August 2012 to April 2013. We used content analysis techniques to analyze the data.

**Results:**

Health professionals’ perceived barriers to cervical cancer care included: (i) patients and community related barriers e.g. lack of awareness on cervical cancer and available services, discomfort with exposure of women’s genitals and perceived pain during pelvic examinations, and men’s lack of emotional support to women (ii) individual healthcare professional’s challenges e.g. inadequate knowledge and skills about cervical cancer management; (iii) health facility related barriers e.g. long distances and lack of transport to cervical cancer screening and care centers, few gynecologists and lack of pathologists, delayed histology results, lack of established palliative care services and inadequate pain control; and (iv) health policy challenges e.g. lack of specialized cancer treatment services, and lack of vaccination for human papilloma virus. Other challenges included increased number of cervical cancer patients and late stage of cervical cancer at presentations.

**Conclusions:**

Operational level healthcare professionals in northern Uganda reported several practical challenges facing cervical cancer care that influence their decisions, management goals and practices. The challenges and proposed remedies can inform targeted interventions for early detection, management, and control of cervical cancer in Uganda.

## Background

Several challenges face cervical cancer screening programs and help-seeking for cervical cancer in sub Saharan Africa. The majority of cervical screening programs are opportunistic and are faced with challenges including poor physical access to cervical screening facilities, low level or lack of knowledge about cervical cancer screening and its benefits [1], low level of self perceived risk for cervical cancer, and understaffed and poorly equipped health facilities [2,3], and long distances to screening facilities and high transport costs [4,5]. Furthermore, inadequate training and few human resources that are poorly distributed affects effectiveness of cervical screening and management in developing nations [6].

Persistent stress related to challenging working environments with cancer patients often leads to burnout and poor quality of care [7] as well as low morale and distress to the healthcare professionals. Likewise, lack of knowledge, misconceptions about disease and lack of skills in management of a disease among health professionals may lead to suboptimal care [8].

There are limited data from operational healthcare providers on barriers and proposed remedies for cervical cancer care, yet the healthcare providers are well placed to contribute context specific strategies that can catalyze policies on cervical cancer early detection and control. Understanding of local factors influencing care is critical to design of targeted interventions [9] to promote help-seeking.

In this study we explored the perceptions of operational level healthcare professionals who work directly with cervical cancer patients on the challenges faced by women seeking cervical screening, cervical cancer diagnosis and management, and challenges faced by health professionals in providing cervical cancer care.

## Methods

### Study setting

This study was conducted in St. Mary’s hospital, Lacor and Gulu Regional Referral Hospital in Gulu district, northern Uganda from August 2012 to April 2013. The two hospitals serve as referral facilities for the whole of the mid northern region. Lacor is a missionary hospital with 482 bed capacity [10]. Gulu hospital is a 335 bed capacity public facility [11]. These two hospitals are the main facilities with capacity to diagnose and provide surgical treatment such as hysterectomy for cervical cancer patients in the mid northern region which include Kitgum, Pader, Agago, Lamwo, Amuru, Nwoya, and Oyam districts. Both Lacor and Gulu hospitals offer cervical screening services. While Lacor hospital had three resident gynecologists and a medical officer during the study period and offered cervical screening with visual inspection with acetic acid/visual inspection with lugol’s iodine (VIA/VILI), Pap tests and colposcopy, Gulu hospital had one medical officer, a visiting gynecologist, and only offered VIA for cervical screening.

### Study design

We chose a qualitative design using key informant interviews because it allows for detailed exploration of personal experiences and thus provide deep insights into the issue under considerations [12,13].

#### Sampling method and respondents

The two hospitals were purposefully selected because they have the capacity to manage cervical cancer and therefore the health professionals at the operational levels would have practical experiences with challenges to and enabling factors for quality biomedical care for cervical cancer. We defined operational level healthcare professionals as qualified health workers including nurses, midwives and medical doctors who during their daily duties directly interface with cervical cancer patients. Help-seeking was defined as all the processes involving decisions to attend cervical screening, symptoms recognition and appraisal, decision to seek help from healthcare professionals, the process of diagnosis and treatment. We purposefully selected the medical directors and other respondents from the gynecology and obstetrics wards and from the cervical screening units of the two hospitals. In Lacor hospital, there were sixteen midwives in the obstetrics and gynecology ward working in turns in the gynecology, maternity, screening sections and labor ward. The ward manager and four other respondents (midwives) were selected, one from each of the sections aforementioned. In addition, a medical officer and the head of obstetrics and gynecology department as well as the medical director, were also selected.

In Gulu hospital, five respondents (midwives and nurses) were selected; three from the gynecology ward and two from the cervical screening unit. The medical director of the hospital and the only medical officer in the gynecology department were also included. The visiting gynecologist to the public hospital is the medical director of Lacor hospital and was already selected.

#### Data collection tool and procedures

A semi-structured key informant interview (KII) guide (Table 1) developed on basis of similar studies was used to collect data. The constructs in the guide captured information on respondent’s gender, professional experience, and their perceptions on cervical cancer patients’ challenges and facilitating factors during help-seeking. During pre-testing the guide, the authors discussed the findings and adjusted the themes so that responses would capture all the intended constructs. The study team approached the medical directors and introduced the study and intended respondents. The medical directors of the two hospitals coordinated the organization of meetings with the head of departments and ward managers to select the suitable respondents. Appointments for interviews were made with the prospective respondents on the dates and hours most suitable to them.

**Table 1:** Health Workers Key Informant Guide

**Categories**	**Themes**	**Probes**
Theme 1	Duration of practice as health worker	Duration of service, highest qualification, employment status
Theme 2	Enabling factors and barrier to diagnosis and management of cervical cancer	Review process of diagnosis of cervical cancer here, factors that positively or negatively influence health seeking and diagnosis and treatment in this hospital, availability of screening, special cancer clinic, Pap smear and in-house pathology services; management challenges and referral, reason for referral and patients “ response to referrals; follow up care
Theme 3	Practical challenges perceived to be faced by cervical cancer patients	Observed challenges, complaints from patients and families, decision making for tests and treatments such as operations to remove uterus, role of husbands, and delays in decisions for operations
Theme 4	Proposed remedies for cervical cancer patients	Problems/challenges facing cervical cancer management which undermine outcome of treatment and ways to improve treatment outcomes

The first author together with three trained research assistants - two men and a woman conducted all the interviews. The research assistants were trained for two days and they participated in the pre-testing of the study tools during the pilot phase of the study project. These research assistants hold bachelor degrees and all of them have had experiences in community surveys, interviews and health related researches with different research organizations.

Interviews started with the nurse ward managers, followed by the heads of departments, the other nurses and midwives, and then lastly the medical directors. Individual interviews were conducted in English in quiet rooms within the hospitals and were audio-recorded.

### Data analysis

Verbatim transcription of the field notes and audio recordings were immediately done following each interview. Before analysis, the principal investigator read all the data transcripts while listening to the audio recordings to ensure completeness, accuracy and correct labeling of the transcripts. Six transcripts were independently read by two of the authors. The two authors thereafter independently formulated emergent codes, and then met, discussed and agreed on the codes to be used in data analysis. Codes were evaluated in the light of the study guides, study objectives and the data.

The individual transcripts were entered into ATLAS.ti version 6.1 as separate primary documents in a new hermeneutical unit. ATLAS.ti was used mainly to code, organize and track changes in the dataset during analysis. Thematic content analysis approach was used in data analysis [14]. Data collection and analysis were done concurrently. Issues that emerged during early interviews and analyses were followed through in subsequent interviews. The individual interviews were the units of analysis. Each primary document was read through line by line and codes were assigned to segments of the data that corresponded to the particular codes. Similar ideas within the data were aggregated into themes and subthemes. Memos were written alongside themes. More boarder and abstract categories emerged as the authors shared their understanding of the data. Verbatim quotations from the texts were used to validate results.

### Rigor and validity in data collection and analysis

The interviews were open ended and respondents were allowed to discuss the questions in an uninhibited manner while being guided to remain focused to the topic of interest. Appropriate probes were used to obtain detail information on responses. Before transitioning to the next theme or statement on the study guide, the interviewer read back the summary of the issues that emerged in the preceding section and participants were allowed time to confirm, add or withdraw any aspect of recorded information they felt did not represent what they had said. Detailed field notes and digital audio recordings were done for all interviews. Interviews were conducted until data saturation was reached [15,16]. In total, we conducted 15 key informant interviews.

### Ethical considerations

This study was approved by the Makerere University School of Medicine Ethics Review Committee (SOMREC) and the Uganda National Council of Science and Technology (UNCST). Additional written permissions were obtained from the institutional review boards of Gulu regional referral hospital and St. Mary’s hospital, Lacor. Each study participant provided signed informed consent before participation in the study. Additional verbal consents to audio record interviews were obtained from each respondent. No participant declined audio recording or participation in the study.

## Results

Four thematic areas on challenges facing cervical cancer care emerged: (i) patients and community related barriers; (ii) individual healthcare professional’s challenges; (iii) health facility related barriers; and (iv) health policy challenges. Across the data, the challenge of increasing number of cervical cancer patients and late stage at presentations was reported.

### Study respondents

Ten women and five men, including 10 nurses/midwives, two gynecologists, two medical officers and one surgeon participated (Table 2). The age of the respondents ranged from 26 to 59 years with a mean of 38.5 years. The academic qualifications of the respondents ranged from certificate in nursing/midwifery to master of medicine in surgery and in obstetrics and gynecology. Some of the respondents possessed other academic qualifications including master of health services management and diploma in public health in addition to their main health qualifications. The duration of services as health workers ranged from 2 to 39 years.

**Table 2:** Demographic characteristic of respondents by qualifications

**Highest health qualification**	**Gender (M/F)**	**Age (years)**	**Duration of services as health worker (years)**	**Order of interview**	**Cadre/Title**
Certificate	F	26	2	R7	Enrolled Comprehensive nurse
Certificate	F	28	5	R8	Enrolled Midwife
Certificate	F	59	35	R14	Enrolled Midwife
Diploma	F	32	8	R1	Registered Nurse/Midwife
Diploma	F	44	19	R2	Registered Nurse/Midwife
Diploma	F	43	22	R3	Senior Nursing Officer
Diploma	F	52	39	R4	Senior Nursing Officer
Diploma	F	30	7	R9	Registered Midwife
Diploma	F	32	9	R10	Registered Midwife
Diploma	F	26	5.5	R15	Comprehensive Nurse
Bachelor degree	M	45	15	R6	Medical doctor
Bachelor degree	M	27	2	R12	Medical doctor
Master degree	M	47	22	R5	Consultant Surgeon
Master degree	M	36	10	R11	Gynecologist
Master degree	M	51	25	R13	Senior Consultant Gynecologist

In general, the respondents from the public hospital had more concerns with inadequate human resources for health, lack of equipments and supplies for proper diagnosis and management of cervical cancer while these concerns were not as strongly expressed by respondents from the missionary hospital where greater emphases were put on quality related challenges such as long waiting times, delayed histology results and other patients-related challenges.

### Challenges and proposed remedies

The challenges to cervical cancer care and the proposed remedies are presented thematically in Table 3 and Table 4 respectively. Verbatim quotations are followed by the respondent’s cadre and years of working experience.

**Table 3:** Challenges to health seeking and cervical cancer care in Gulu

**Challenges in cervical cancer care**	**Respondents by hospital and gender**				**N**
	**Public hospital**		**Mission hospital**		
	**Male**	**Female**	**Male**	**Female**	
**A. Individual and community related challenges**					
Lack of awareness amongst women; Inadequate sensitization and mobilization of communities	R5	R1, R3, R4	R11	R7, R8, R9	8
Late stage disease at presentations and its management challenges	R5, R6	R2, R3, R4, R14	R11	R7, R8, R10, R15	11
Inadequate psychosocial support to and abandonment of patients by relatives	R6			R7, R10, R15	4
Fear of exposure (undressing and lying in lithortomy position) and perceived pain from speculum during screening		R3	R13	R15	3
Complaints against/Stigma from other patients because of smell from cervical cancer patients		R3			1
Patients cannot pay for services such as histology and EUA	R6		R11	R10, R15	4
Refusal to consent				R7, R9, R15	3
Inadequate contact address of patients and hence poor follow up to let results known to them	R5		R11		3
**B. Individual health professional related challenges**					
Inadequate knowledge and skills in cervical screening, cervical cancer diagnosis and care	R5, R6	R1, R2, R3, R14	R10, R11, R12, R13	R7, R8, R15	13
**C. Health system related challenges**					
No pathologist and no Pap smear test	R5, R6	R1		R15	4
No gynecologists or doctors in gynecology department to supervise screening, do biopsy, do operations	R5, R6	R1, R2, R4			5
Delayed histology results and or specimens/results getting lost	R6	R2, R3		R10, R15,	5
Inadequate space/ward for cervical cancer patients		R3	R12		2
Abandonment of histology results	R5		R11		2
Long distance to and lack of transport to screening points		R4, R14		R10	3
Congestion at screening, long waiting time to screening, scheduling delay			R11	R7	2
Lack of formalin for biopsies		R2			1
Few nurses and midwives to support screening and health talk to women		R3, R14		R15	3
Lack of blood for transfusion before and during operations			R12	R15	2
Stock out of morphine and inadequate pain control				R7, R10	2
Hospital does not provide food to the patients who stay for long and run out of money and support	R6	R3, R4, R14		R10	5
Inadequate labeling of clinics and lack of integration of services	R5				1
Poor reputation of hospital and inhibiting health seeking there	R5				1
Men health workers doing screening				R7, R15	2
**D. Health policy related challenges**					
Lack of vaccination against HPV	R5	R1, R2	R11,R12, R13	R15	7
Radiotherapy is very far and patient cannot afford transport and cost of care in city	R6	R14			2

Note: R1, R2 = Respondents’ order of interview as on Table 2.

**Table 4:** Proposed remedies to cervical cancer health seeking challenges in northern Uganda in decreasing order of frequency

**Proposed remedies for cervical cancer care**	**Respondents by hospital and gender**				**N**
	**Public hospital**		**Mission hospital**		
	**Male**	**Female**	**Male**	**Female**	
Sensitization and community mobilization	R5, R6,	R1, R2, R3, R4	R11, R12, R13	R7, R8, R9, R10, R15	14
Establish regional cancer units to improve cancer care	R5, R6	R2, R3, R14	R11, R12, R13	R7, R8, R9, R10, R15	13
Devolution of cervical cancer screening services to lower health units	R6	R1, R2, R3, R14	R12, R13	R8, R9, R15	10
Establishing regular screening outreach programs for cervical cancer screening	R5, R6	R1, R3, R14		R7, R10, R15	8
Posting more gynecologists and medical officers to the gynecology departments to provide care to women	R5, R6	R1, R2, R3, R14		R15	7
Regular continuous professional development (CPD) sessions on cervical cancer		R1, R3, R14	R11, R12, R13	R8	7
Lack of radiotherapy services to manage advanced cervical cancer	R6	R2, R3	R11, R12	R7, R10	7
Establish pathology services in the region	R5, R6	R1	R12, R13	R15	6
Primary prevention through rolling out HPV vaccination	R5	R1, R2	R11, R12, R13		6
Prioritization and training of more health human resources	R5, R6			R8, R10, R15	5
Involve men in women’s health matters		R2, R3		R8, R15	4
Provide food to cervical cancer patients		R3, R4, R14		R10	4
Establish palliative care services and maintain constant supply of morphine in the region		R1, R3	R11	R9	4
Girl child education and incorporation of topics on cervical cancer in primary school education curriculum		R4	R13	R9	3
Advocacy	R5	R3		R9	3
Improve on blood supplies to hospitals			R12	R15	2
Recreational and psycho-relaxation activities		R3		R10	2
Strengthen referral system to the Uganda Cancer Institute	R5, R6				2
Increase funding for health	R5		R11		2
Establish a population cancer registry in the region			R11		1

Note: R1, R2 = Respondents’ order of interview as on Table 2.

### Patients and community related challenges

The majority of respondents reported challenges that included lack of awareness and knowledge on cervical cancer and available services, inability to pay for screening and diagnostic services, invasion of privacy during pelvic examinations, inadequate psychosocial and family support to patients, and patients’ perceived stigma related to foul smelling vaginal discharges.

#### Lack of knowledge and awareness about cervical cancer and available services

The women were perceived to be unaware of cervical cancer symptoms, risk factors, and available services for the disease; the women would thus wrongly interpret and attribute the symptoms of cervical cancer to other conditions. Subsequently, they engage in treatment for those perceived conditions and end up reporting to hospitals late when cervical cancer is advanced and treatment outcomes poor.

*“. . . you know, it is only when the women know about how cervical cancer presents that they can come to the hospital early and we make the diagnosis and manage them”, ****(Gynecologist, 10 yrs)***

Women reportedly have inadequate knowledge of cervical cancer symptoms, course and progression. This was thought to influence their help-seeking behavior. Respondents thought that women would report earlier if they knew the symptoms of cervical cancer and believed in the effectiveness of modern medicines in management of cervical cancer.

*“The women do not have correct ideas about the cancer. Patients come not knowing they have cervical cancer”,* ***(Registered Nurse, 8 yrs)****.*

Lack of awareness as a barrier was cited by almost all the respondents irrespective of their level of training and hospital of practice, and respondents suggested community awareness campaigns using mass media including the FM radios as a remedy.

*“For the way forward, we can have radio programs; the government can sponsor people to go on the radio and have discussions with the community. We also need to do outreaches on cancer and we tell people where to go and what signs to look out for,”* ***(Comprehensive Nurse, 5.5 yrs)****.*

However, some respondents were concerned that radios may not reach the target group - the women.

*“We have to use appropriate means for reaching mothers; things like radios are a property of a man in a home, and have been found to not be a good way of giving messages for women in the villages. In the villages, men own the radios and women do not have access to the radios*”, **(*Registered Nurse*/*Midwife*,** ***19 yrs*)**.

#### Late stage cervical cancers

The majority of cervical cancer patients reportedly present very late when the available treatment modalities cannot help. Late presentations occur for several reasons;

“*They say they come late because of lack of money*; *on the other hand they do not suspect any problem when they are bleeding because women bleed throughout their lifetime. They may relate even abnormal bleeding with their usual bleeding* - *even if they are above the age of menstruation. But also most health workers miss the diagnosis and giving treatment at early stage*, *while most hospitals lack medical officers with specialized skills to provide the specialized services*,” **(*Senior Consultant Gynecologist*,** ***25 yrs*)**.

Health workers in the peripheral units were reportedly not well equipped to screen and diagnose cervical cancer and may contribute to delaying diagnosis of cancer.

“*What I have seen is that the patients first go to the clinics and medical centers where there are no such things as cervical screening for early detection. Also*, *in the clinics*, *when someone comes may be with like bleeding*, *health workers mistake it for something else and he*/*she treats that. By the time the patients come here*, *the cancer is in late stage*,” **(*Comprehensive Nurse*,** ***5*.** ***5 yrs*)**.

On the other hand, patients reportedly do not attribute the symptoms they get to cervical cancer but to other common diseases for which they seek care in small health units, clinics and drug shops which are operated by personnel who are not competent to make diagnosis of and manage cervical cancer. In addition, lack of money may prevent patients from promptly seeking care in hospitals with symptoms.

“*I interviewed some cervical cancer patients. I found that they have always ignored the early signs such as post coital bleeding and discharges and when you ask them*, *they say* ‘*well I did not know it is a sign of a big problem*.’ *Poverty also makes people go to small health units where those running cannot even detect cancer*,” **(*Consultant Surgeon*,** ***22 yrs*)**.

#### Perceived inappropriate exposure during pelvic examinations

Respondents reported that patients were sometimes deterred from seeking care in hospitals because they would be asked to undress completely, to lie in the lithotomy position (lying on the back with the legs put up and spread apart) and because they were concerned about pain from internal examinations.

“*Women do fear exposing their private parts during examinations. That is the fear of undressing themselves and undergoing examination. They also have a fear that the vaginal speculum is very painful*”, **(*Senior Nursing Officer*,** ***22 yrs*)**.

#### Age and gender differences between patients and health workers

Respondents reported that some older women do not like to undress and have their genitals examined by young doctors, particularly young male doctors. They would prefer an older doctor, especially a female doctor to do cervical screening. Even some health workers in the study hospitals reported that they had not had cervical screening because they did not wish their male counterparts to conduct intimate examinations when they do not have any symptoms and there is no risk to life in contrast to pregnancy and delivery when they did not mind male doctors examining them.

“*If it is an elderly patient she may not accept the young person to look at her so you find it difficult to convince this lady to get her up the coach and undress*; *to get her undress when the doctor is a male. But if it is the youths*, *they are easy*,” **(*Comprehensive Nurse*,** ***5*.** ***5 yrs*)**.

This view was shared by the senior doctors as well;

“*They say when they come to hospitals they meet their sons*, *and daughters. They feel embarrassed to undress before these young doctors*”, **(*Senior Consultant Gynecologist*,** ***25 yrs*)**.

#### Inadequate emotional and financial support for women

Men’s lack of emotional and financial support to women reportedly hinders both cervical screening services and early help-seeking with symptomatic cervical cancer and other diseases affecting women.

“*The men should know the health issues concerning women*; *they should know that this can be a problem to my wife*. . . . *sometimes instead of going to screening*, *they want the wives to be going to the garden* - *so they have to be made aware that this is a serious issue and if the service is there*, *you allow your woman to go*! *Or if it is very far*, *then ride her on that bicycle*! *Men should support their wives. They should provide transport*, *give them permission to go screen and should give them the money needed*; *they should also accompany the wives to the screening centers*,” **(*Registered Nurse*/*Midwife*,** ***19 yrs*)**.

Awareness programs should target men as well so that they have more knowledge about cervical cancer;

“*Men need to be sensitized on issues of cervical cancer so that they do not look at children as source of wealth alone but also the risks it carries with it. They should be talked to about this issue of many and frequent child bearing which to men is a source of wealth. In this way they will also help the women fight cervical cancer*”, **(*Enrolled Midwife*,** ***5 yrs*)**.

Cervical cancer patients are reportedly often abandoned in the hospitals by relatives.

“*The cervical cancer patients also face neglect by their relatives*; *sometimes when they are having offensive discharges and they have broken the news to the relatives*, *for them they just say* – ‘*aah this one is just going to die*, *why should we care about*!’ *They end up leaving that patient in the hospital. Maybe because of the fear of the smell*, *they don*’*t want to stay nearer to the patient*; *the patient is just left alone*” **(*Enrolled Comprehensive Nurse*,** ***2 yrs*)**.

The belief that cervical cancer is contagious and the prolonged course of cervical cancer were perceived to sometimes explain in part relatives’ abandonment of patients.

“*I see these patients are many times neglected by their relatives especially if their cancer is already advanced. Most of the relatives think cervical cancer can transfer when they are nursing the patients. But you know this illness also takes long*, *so when people are nursing you for long people get tired*,” **(*Enrolled Midwife*,** ***35 yrs*)**.

#### Refusal to consent

Three respondents reported circumstances when cervical cancer patients with early stage operable cancers refused to provide written informed consents for operations to remove their uteri (hysterectomy), with associated significant delays in providing the required care.

“. . . *when they come sometimes they don*’*t want operation. They are like*, ‘*I don*’*t have a child or let me first consult my husband*.’ *Last week there was a forty five years old lady who refused hysterectomy. When we explained to her that the operation is to remove her uterus*, *she said* ‘*no*, *no*, *no. I don*’*t have a child*, *how can you remove my uterus*?’ *I have seen so far three such cases in the last six months*,” **(*Comprehensive Nurse*,** ***5*.** ***5 yrs*)**.

However, the patients reportedly eventually accept the hysterectomies after discussions with their husbands and family members.

### Individual health care professional challenges

The majority of respondents reported that many health professionals, mainly in the lower level health facilities lack specialized training, knowledge and skills for cervical screening; lack the clinical astute to diagnose cervical cancer early based on symptoms and signs.

#### Inadequate knowledge of healthcare professionals on cervical cancer and care

In both study hospitals respondents were concerned that their colleagues in the other hospitals and lower level health facilities lacked up-to-date knowledge on cervical cancer and the management options.

“*The knowledge is lacking across the board. The study of Mutyaba in Mulago hospital showed that health workers have very poor knowledge of screening for cervical cancer. But you know even some gynecologists do not have the correct knowledge on cervical cancer screening and management*! *I can count for you gynecologists in this country who can do cryotherapy and LEEP well*!” **(*Gynecologist*,** ***10 yrs*)**.

The problem of cancer prevention and management in Uganda needs more than just continuous professional development; it requires training of more oncology doctors and nurses so that the workloads related to cancer screening, diagnosis and management can be reduced.

“*Then I think more people should study more about cancers. In that case*, *the diagnosis of cancer will be faster. It would also reduce the work load*, *more cases are seen*, *more cases are treated and then in total you have more time with the patient than when you have few gynecologists*,” **(*Comprehensive Nurse*,** ***5*.** ***5 yrs*)**.

Similarly, a senior midwife reiterated training need;

“*And also I think more health workers should be trained*; *in particular the health workers should be trained on screening*; *even those in the villages* (*rural health centers*) *so that in every health center all the health workers have the skills and they can mobilize the community for screening so that the people who have cancers are identified early enough*,” **(*Enrolled Midwife*,** ***35 yrs*)**.

### Hospital and health system barriers

Long distances to health facilities and associated transport costs, few gynecologists in the region, lack of pathology services, lack of chemotherapy and radiotherapy services were reported barriers to help-seeking and quality care for symptomatic cervical cancer patients.

#### Long distances and associated transport difficulties to health facilities

A barrier frequently mentioned by more than half of the respondents is the long distances patients travel to the few screening and care facilities. They do not only need money to transport themselves but also ample time. Respondents thought that competing commitments such as gardening may compromise time for screening and delay help-seeking.

“*Long distances to Gulu town where screening services are available* . . . *rural women may not have money and they have to take time off gardening to come to town on those specific days when screenings are done in Gulu and Lacor hospitals. Even though they hear about screening*, *they will not reach may be due to long distances*; *and inadequate knowledge of services offered by these hospitals since the hospitals are far away from them*”, **(*Registered Nurse*/*Midwife*,** ***8 yrs*)**.

Impassable roads, long distances and lack of money to pay for transport compound the transport difficulty.

“*I see there is problem of transport*. . .*There is no money and also because the place is far. The road is also bad especially when it rains*,” **(*Enrolled Midwife*,** ***35 yrs*)**.

#### Lack of finances for screening and cervical cancer management

Access to care was also viewed from economic perspectives and convenience to clients.

“*High charges for cervical cancer services*; *this prevents some people from coming for the tests. Patients complain of the 5*,*000 shillings* (*2USD*) *they pay to be checked*”, **(*Registered Midwife*,** ***7 yrs*)**.

Free cervical screening might circumvent high cost of services which the majority of women cannot afford;

“*Maybe the government can take charge and pay for the services. That is*, *to provide free cancer screening and care*,” **(*Comprehensive Nurse*,** ***5*.** ***5 yrs*)**.

#### Few gynecologists and lack of pathologists

Timely and quality biomedical care for cervical cancer may require the presence of gynecologists, in-house pathology services and planned follow up care among other requirements.

“*As I talk now there is no any gynecologist in Gulu regional referral hospital. The absence of gynecologists and other specialized doctors delay the process of decision making and that gives room for cancer to grow*”, **(*Senior Nursing Officer*,** ***22 yrs*)**.

Lack of gynecologists and cancer specialists in the region reportedly account in part for low quality of care, delays in decisions, diagnoses and offering appropriate services, and subsequent poor treatment outcome.

“*Sometimes there is delay in diagnosis and operation. For those diagnosed early*, *if a gynecologist were present*, *operations would be done and that would make us get better results*.. . . *Also the cadre of health workers doing the VIA may also make them miss the diagnosis. There should be a gynecologist to supervise and help them with uncertain cases. These lower cadres may misdiagnose cervical cancer as not cancer and the person goes back home with a cancer. There is also a colposcope at the screening center* - *but there is no one to use the machine*! ”, **(*Medical Officer*,** ***15*** **yrs).**

During the study period, there were no resident pathologists in both study hospitals and the region. The respondents were concerned that the lack of in-house pathology services has lead to delay in getting cytology/histology results, losses of biopsy samples and or results with subsequent frustrations to the women and their families who may have to travel to pick their results more than twice.

“*Without Pap smear you cannot detect much cancer at an early stage* . . . *you cannot conclude diagnosis*.”, **(*Registered Nurse*/*Midwife*,** ***8 yrs*)**.

#### Delayed histology results

Respondents were concern with the repeated delay or even loss of histology/cytology results.

“*Sometimes the histology takes up to a month*; *patients come and find no results. Sometimes samples get lost* - *usually because they are left lying in theater for quite a long time and someone else can mishandle them. Sometimes results get lost and you may have to repeat the biopsy*”, **(*Registered Nurse*/*Midwife*,** ***19 yrs*)**.

When results delay and or get lost, some clients reportedly may think that they are actually positive for cervical cancer but the health professionals are only withholding the truth from them, and so they get distressed and sometimes never come back for such results.

#### Lack of blood for transfusion

Two respondents, a nurse and a medical officer emphasized the inevitable delays that often occur in diagnostic and staging procedures such as examination under anesthesia (EUA) because of lack of blood for transfusion.

“*Some of the cervical cancer patients come when they are dehydrated*; *when they have over bled*, *and they are anemic. So you first have to deal with the anemia and dehydration. But it is very difficult to get blood. Almost every month we experience the problem of lack of blood. Sometimes you reach a point where you have to bring the attendants to donate*, *which is said to be unacceptable but you have to do it to save the life of the patient*,” **(*Comprehensive Nurse*,** ***5*.** ***5 yrs*)**.

#### Lack of established palliative care services and inadequate pain control

Two respondents, both nursing staff, were concerned about the frequent lack of oral morphine and the relentless pain that cervical cancer patients undergo.

“*Majority of patients prefer morphine but sometimes the drug is lacking or out of stock. We had two patients here that refused codeine and insisted they wanted the other liquid pain killer*”, **(*Registered Midwife*,** ***7 yrs*)**.

#### Lack of food for patients

Some respondents were concerned about inadequate nutritional support by the hospitals to the cervical cancer patients who generally are undernourished, stay in hospitals for long and often deplete their food supplies.

“*These patients usually lack nutrition*; *they are anemic. Patients may not have anything to eat and the hospital also does not provide food yet the cervical cancer patients are very poor and they spent long time in the hospital*”, **(*Senior Nursing Officer*,** ***22 yrs*)**.

### Health Policy challenges

Respondents in both hospitals were concerned with the lack of enabling policies on key services such as devolution of specialized cancer services to regional hospitals and national roll out of HPV vaccination.

#### Lack of specialized cancer treatment services and long distance to Mulago

More than two thirds of the respondents were concerned with the lack of radiotherapy and chemotherapy services needed for the care of cervical cancer and other cancer patients in the countryside who spend lots of money to travel to the city for specialized cancer services from Mulago radiotherapy department and the Uganda Cancer Institute (UCI). Such services are supposed to be free or subsided by government so that patients can afford.

“*The women with cancers who missed to screen and who now have advanced diseases face a big problem*; *we need to get for them drugs that can push them somewhere at least. Chemotherapy and radiotherapy should be made available. Radiotherapy should not only be in Mulago. The centers should be many. Let these services be extended into the regional hospitals at least. And they should be cheaper because currently they are not free and that is one reason why patients are not going to Mulago for the radiotherapy. Government should provide free chemotherapy like ART is being given free to HIV clients*”, **(*Registered Nurse*/*Midwife*,** ***19 yrs*)**.

Some cervical cancer patients who are referred for specialized cancer care to Mulago reportedly do not actually go because the patients and families face challenges including unaffordable cost of transport, investigations, and medicines while in Mulago.

“*When these patients are referred to Mulago hospital many of them refuse to go because they do not have money for feeding and transport. They also may have fears because they will find strangers there*; *but when they are in Gulu they can see people from home*; *people from home can come and visit them but if they are now very far*, *that will be very difficult*,” **(*Enrolled Midwife*,** ***35 yrs*)**.

Devolution of specialized cancer services would mitigate a number of challenges patients from upcountry stations in Uganda face as they travel to the city for specialized care. Devolution of cancer services will also ease on the congestions at the cancer centers in the city.

“*For me basically*, *I would want not to speak about northern Uganda only but the whole of Uganda. We have only one place for treating cancer*; *why don*’*t they also regionalize it* – *east*, *west*, *central*, *north* - *so that we also have a center for northern Uganda*? *That will also avoid overcrowding in Mulago. I think it will also reduce on transport cost*,” **(*Comprehensive Nurse*,** ***5*.** ***5 yrs*)**.

#### Lack of vaccination against the human papilloma virus (HPV)

Respondents discussed the lack of policy on the inclusion of the HPV vaccine into the national immunization program and rolling out of the vaccination program to all districts. Respondents noted that this vaccine is too expensive for most rural community members to afford from the private sector.

“*It has taken government about 5 years* (*since 2008*) *to roll out the vaccination program from the 2 pilot districts. The vaccination is primary*; *am putting it number last* - *not because it is not important but rather because of two reasons* - *the vaccine is expensive but also because the vaccines available can control only 2*–*3 types of high risk HPV subtypes*, *and so vaccination cannot replace the other methods of prevention because it is not 100* % *anyway*,” **(*Gynecologist*,** ***10 yrs*)**.

## Discussions

In this study, the operational level healthcare professionals in the obstetrics and gynecology departments and the medical directors of the two study hospitals pointed out a number of patient and community related challenges, individual health professional inadequacies, health system related barriers, and health policy related barriers to help- seeking for cervical cancer in northern Uganda.

Lack of awareness and knowledge about cervical cancer symptoms, available services and services locations, and the lack of knowledge on the benefits of cervical screening were critical perceived barriers for cervical screening and or help-seeking for cervical cancer symptoms. The low health related knowledge reported in this study maybe related to the general low literacy rate of 48. 8% among women in the study region as compared to 63.3% and 90.6% in western Uganda and Kampala respectively [17]. However, limited knowledge about cervical cancer was also reported in Serbia [18] and Botswana [19]. Increasing women’s health literacy through community health education may improve cervical cancer screening uptake and early help-seeking for symptomatic cervical cancer.

In this study, discomfort attributed to the exposure of women’s genitals, the position in which women lie when health professionals do pelvic examinations and take samples during screening and diagnosis of cervical cancer and the perceived pain from the instrument (speculum) inserted during pelvic exams were reported as challenges to cervical screening and help-seeking for cervical cancer symptoms. In a recent study in central Uganda, 155 of 384 women declined cervical screening because of concerns with invasion of their privacy, and fear of pain or discomfort associated with the cervical screening procedure [20]. Concerns about privacy, discomfort with pelvic examinations and sacredness associated with women’s genitals were also barriers to care seeking among Maori women in New Zealand [21] and Arab women in the Emirates [22]. To increase cervical screening uptake and encourage early help-seeking for symptoms of cervical cancer, health systems might need to consider socio-cultural beliefs about the sacredness of women’s genitals and the degree of exposure during examinations as well as gender of the health professionals when planning cervical screening and gynecology services in low- and middle-income countries (LMICs) such as Uganda.

A critical barrier to care is the abandonment of cervical cancer patients by their relatives in the hospitals because of the bad smell of vaginal discharges from the patients, difficulty in keeping good hygiene for the patients who themselves are debilitated and helpless, and fatigue due to prolong hospital stay with associated depletion of resources. Abandonment of cervical cancer patients by relatives and poor attitudes of health professionals about cervical cancer patients were also reported by health professionals in Kenya [23]. Public health education programs in communities may need to include accurate information about the causes, symptoms and prognosis of cervical cancer so that the negative attitudes and stigma associated with cervical cancer as well as patient abandonment may be reduced or prevented. A *“Be nice to your relative and neighbor when she’s smelly campaign”* might create a sense of solidarity and compassion between cervical cancer patients and family and friends.

Lack of financial and emotional support from men and denial of permission for women to go to hospital for early help-seeking for symptomatic cervical cancer featured frequently in this study. It is thought that men might not support the women because of men’s ignorance of the benefit of cervical screening and early help-seeking for symptomatic cervical cancer. Therefore, deliberate policy to involve men in women’s health matters might improve the health of women in Uganda and other LMICs where domestic power balance is skewed towards the men. Indeed, cervical cancer programs that involved men and community members have been shown to gain more acceptability and uptake [24].

Respondents reported that many health professionals are not conversant with cervical cancer symptoms and signs. In Kenya, health professionals from regional and national referral hospitals reported inadequacies in training on management of patients with cervical cancer [23]. Cervical cancer patients perhaps present late in hospitals not because they had never had any contacts with health workers before but because lower level health workers did not suspect cancer and were providing other treatments, e.g. for sexually transmitted infections (STIs). Health professionals’ lack of clinical skills and associated late stage cervical cancer was reported in South Africa [25]. Chirenje et al also found that health care professionals lacked knowledge and skills necessary for proper management of cervical cancer [3]. Some respondents reported that health professionals may also avoid doing adequate vaginal and speculum examinations and simply treat women based solely on symptoms. Physicians, general medical officers, paramedics, midwives and nurses need to be aware of the need and to perform digital vaginal and speculum examinations. A study to assess the attitude and quantify the magnitude of such non gynecologists in performing vaginal and speculum examinations may shine light onto the root causes for non-performance of the examinations. Perhaps retraining and reorienting qualified health care professionals through continuous professional development might update their knowledge and skills in cervical cancer care. In addition, short term fellowships in cancer prevention and management for qualified doctors and gynecologists could also increase the critical mass of health professionals with the required knowledge and skills in Uganda and other LMICs that face shortages of qualified health professionals for cancer care.

Only few health facilities in the post conflict northern Uganda can provide cervical screening, diagnosis and surgical management of cervical cancer; all of them are located in Gulu town. Women therefore have to find time off their busy schedules with gardening and family chores, get permissions from their husbands, secure transport money and then travel to the town where screenings and diagnoses take place. The competing demands on the women’s times might constitute hindrances for screening. In addition, this region has just emerged from a 20 years old civil conflict that had restricted the population in internally displaced people’s (IDP) camps and deprived them of viable economic activities [26] and therefore money for transport and screening may be insurmountable challenges. Long distances to service centers [4], poor transport systems [22] and lack of money for transport and services fees [23,27] have been reported as obstacles for cervical cancer screening and care elsewhere too. Policies to take cervical screening and cervical cancer diagnostic and treatment services nearer to the population might improve access to cervical cancer services in Uganda and other LMICs. In addition, a policy of training health professionals in lower level health units to do cervical screening is needed. Health systems in Uganda and other LMICs might need to strengthen referral patterns to ease movement of patients from lower to higher levels of care.

In this study, respondents reported inadequate number of gynecologists and pathologists and the associated delay of services, inaccurate diagnoses, inappropriate decisions and probably inappropriate treatments. Poor quality services maybe a reason for women not to attend care in such hospitals in the future. Trained human resources for health, particularly pathologists, is lacking in Uganda and many other developing countries [28,29]. Policy to train and retain more gynecologists, pathologists and other cancer specialists may lead to improved quality care and increase service accessibility.

Another barrier to quality care was the frequent lack of oral liquid morphine which leaves cervical cancer patients suffering from uncontrolled pain. In the management of advanced cancers, the provision of organized palliative care with counseling of both patients and families are essential [30]. Governments and other stakeholders in sub Saharan Africa might want to strengthen health facilities and medicines deliveries in particular so that patients with chronic diseases do not suffer avoidable pain and distress because of frequent lack of medicines.

Lack of food as a barrier to help-seeking seems to be overlooked by the hospital management and government. Respondents reported that the hospitals do not provide food for the debilitated cervical cancer patients who require adequate nourishments and energy to withstand side effects of treatments. When patients become starved while in hospitals, they most likely request for discharges and or simply abandon hospitals and go home where they can at least get some food. In a recent study in Pader, a neighboring district to Gulu, immediate needs such as food, safe water and shelter were given priority than health needs such as prevention of HIV infections [31]. Similar findings were reported in Russia, where a tuberculosis (TB) program plunged into chaos because of lack of food for the TB patients [32]. Governments and development partners in LMICs may need to consider food requirements of patients and marginalized communities to increase effectiveness of health and development programs.

The absence of radiotherapy (RT) was reported as a major challenge to cervical cancer care. Post operative chemo-radiation has been shown to be tolerable and improve survival for cervical cancer patients [33-35]. However, in Uganda, there is only one functional cobalt-60 RT machine located in the capital city Kampala [36,37], and the patients travel long distances to get these services. Our study revealed that patients with late stage cervical cancer who require chemo-radiation resent further referral to Kampala because of lack of finances and fear of the unknown in a foreign land where they had never been and where most of them have no relatives to provide support and care. Not only do the absence of RT and frequent lack of oral morphine leave the patients with uncontrolled bleeding and pain respectively but also might discourage patients with late stage diseases from seeking care in the hospitals because they believe that they will still suffer relentlessly despite being in the hospitals. Specialized cancer services need to be taken nearer to the populations; however, there is a lack of policy on devolution of cancer services to regional levels.

Furthermore, respondents were critical on the absence of policy on universal HPV vaccination as primary preventive measure against cervical cancer. In Uganda the vaccine has not yet been incorporated in the routine immunization package so as to allow wider coverage [38]. The biggest barrier to the adoption of the HPV vaccines into national immunization programmes has been the high costs of the vaccines [39]. Uganda government may wish to collaborate with World Health Organization (WHO), Global Alliance for vaccine and Immunization (GAVI) and other development partners to make the HPV vaccine available to the girls who need them.

### Study limitations

This qualitative study explored in details healthcare professionals’ perceptions of barriers and remedies to cervical cancer care, but the study was by no means exhaustive; estimation of the extent of the reported barriers and remedies cannot be assessed by this design. Second, we did not include the health professionals working in the lower level health units where the ability to screen and confirm diagnosis of cervical cancer is currently lacking. These categories of health professionals are however nearer to the community and may have some unique perceived barriers experienced at the lower levels of health seeking. Future studies may need to include a broader area of the country and apply both qualitative and quantitative approaches in order to provide exhaustive evidence to inform policy.

## Conclusion

Findings from this study reveal some of the salient challenges that undermine cervical screening and early help-seeking for cervical cancer symptoms with subsequent late diagnoses of cervical cancer in northern Uganda. The use of qualitative approach allowed us to explore in details the perspectives of operational level health professionals on the nature of the challenges that women encounter when they seek care for symptoms suggestive of cervical cancer. The suggested remedies may guide policy makers on formulating targeted interventions to promote cervical screening, and early detection and prompt management of early cervical lesions and invasive cervical cancer.

## Competing interests

The authors declare that they have no competing interests.

## Authors’ contributions

ADM and HW were involved in study design, development of data collection tool and data collection and analysis. HMK participated in data analysis and manuscript writing. All authors read and approved the final manuscript.

## Pre-publication history

The pre-publication history for this paper can be accessed here:

http://www.biomedcentral.com/1471-2296/14/193/prepub
